# Assessment of Mutations Associated With Genomic Variants of SARS-CoV-2: RT-qPCR as a Rapid and Affordable Tool to Monitoring Known Circulating Variants in Chile, 2021

**DOI:** 10.3389/fmed.2022.841073

**Published:** 2022-02-25

**Authors:** Jenniffer Angulo, Constanza Martinez-Valdebenito, Catalina Pardo-Roa, Leonardo I. Almonacid, Eugenia Fuentes-Luppichini, Ana Maria Contreras, Constanza Maldonado, Nicole Le Corre, Francisco Melo, Rafael A. Medina, Marcela Ferrés

**Affiliations:** ^1^Departamento de Enfermedades Infeciosas e Inmmunologia Pediatricas, Escuela de Medicina, Pontificia Universidad Católica de Chile, Santiago, Chile; ^2^Infectious Disease and Molecular Virology Laboratory, Red Salud UC-Christus, Santiago, Chile; ^3^Advanced Interdisciplinary Rehabilitation Register – COVID-19 Working Group, Faculty of Medicine, Pontificia Universidad Católica de Chile, Santiago, Chile; ^4^Molecular Bioinformatics Laboratory, Department of Molecular Genetics and Microbiology, Faculty of Biological Sciences, Pontificia Universidad Católica de Chile, Santiago, Chile; ^5^Institute for Biological and Medical Engineering, Schools of Engineering, Medicine and Biological Sciences, Pontificia Universidad Católica de Chile, Santiago, Chile; ^6^Icahn School of Medicine at Mount Sinai, New York, NY, United States

**Keywords:** SARS-CoV-2, variants of concern (VOCs), variants of interest, variants under monitoring, RT-qPCR-based surveillance

## Abstract

Since the first report of SARS-CoV-2 infection in humans, the virus has mutated to develop new viral variants with higher infection rates and more resistance to neutralization by antibodies elicited after natural SARS-CoV-2 infection or by vaccines. Therefore, rapid identification of viral variants circulating in the population is crucial for epidemiological assessment and efforts to contain the resurgence of the pandemic. Between January and November 2021, we performed a large variant RT-qPCR-based screening of mutations in the spike protein of 1851 SARS-CoV-2-positive samples derived from outpatients from the UC-Christus Health Network in Chile. In a portion of samples (*n* = 636), we validated our RT-qPCR-pipeline by WGS, obtaining a 99.2% concordance. Our results indicate that from January to March 2021 there was a dominance of non-identifiable variants by the RT-qPCR-based screening; however, throughout WGS we were able to identify the Lambda (C.37) variant of interest (VOI). From March to July, we observed the rapid emergence of mutations associated with the Gamma variant (P.1), which was quickly replaced by the appearance of a combination of samples harboring mutations associated with the Delta variant (B.1.617.2), which predominated until the end of the study. Our results highlight the applicability of cost-effective RT-qPCR-based screening of mutations associated with known variants of concern (VOC), VOI and variants under monitoring (VUM) of SARS-CoV-2, being a rapid and reliable tool that complements WGS-based surveillance.

## Introduction

Since coronavirus disease 2019 (COVID-19) was declared a pandemic in March 2020, severe acute respiratory syndrome coronavirus 2 (SARS-CoV-2) has spread and evolved, disseminating to more than 200 countries, causing more than 265,000,000 of cases and more than 5,000,000 of deaths (1). The continuous circulation of SARS-CoV-2 throughout the world has allowed the virus to continue evolving and to acquire novel genetic mutations (2). In some cases, these mutations provide adaptive advantages to the virus, which is why new viral variants have emerged and outcompeted ancestral strains (2, 3). Currently, a major concern is the emergence of SARS-CoV-2 variants, which have challenged governments and public health authorities to face new pandemic waves worldwide (4). The alarm lies on the potential increase in transmissibility, higher disease severity, re-infections risk, lower vaccine efficacy, and impaired effectiveness of treatments and diagnostic tools (2, 3, 5). A consensus nomenclature was created by the World Health Organization (WHO) to highlight these concepts, categorizing new variants as variants of concern (VOC), variants of interest (VOI) and variants under monitoring (VUM) depending on their epidemiological and morbidity-lethality impact (6). Mutations in the S gene of SARS-CoV-2, which impact the receptor-binding domain (RBD), are of foremost interest since they could modify the affinity for the cellular receptor (7), modify its infectivity, and result in immune evasion (5, 8, 9). The emergence of variants with heightened transmissibility can result in a rapid increase in community cases, generating higher numbers of severe COVID-19 and exceeding the capacities of health care centers (10, 11). For example, the Alpha variant (B.1.1.7) had increased transmissibility compared to the ancestral strain, but the mutations in spike protein did not significantly alter the neutralization capability of antibodies like the Beta (B.1.351), Gamma (P.1) and Delta (B.1.617.2) variants, which have been proven to be less susceptible to antibody neutralization (12–15). While genomic sequencing through whole genome sequencing (WGS) is undoubtedly the most precise and effective tool to trace specific changes inherent to viral evolution, it is particularly expensive to implement for low-and-middle incomes countries where the resources are mainly focused on diagnosis and treatments (12, 16). As long as variants of interest continue to emerge over time, WGS-based approaches are crucial for the identification and to study the evolution of novel SARS-CoV-2 variants (17). To maximize the efficiency of sequencing efforts and resources, an initial assessment of known mutations associated with SARS-CoV-2 variants through reverse transcriptase quantitative polymerase chain reaction (RT-qPCR)-based assays might be useful to discriminate between VOC/VOI/VUM and unknown variants and could better direct the use of WGS for those samples without a definitive variant diagnosis (12). Furthermore, several studies have reported the use of commercial kits to screen VOC using RT-qPCR-based assays for individual assessments (12, 17–22).

In Chile, genomic surveillance of SARS-COV-2 variants allowed reporting the introduction of the B.1.1.7 lineage (Alpha, α) in December 2020, and subsequently the introduction of the P.1 (Gamma, γ) and P.2 lineages (Zeta, ζ) from Brazil and the detection of cases of the B.1.351 lineage (Beta, ß), B.1.427/9 (Epsilon, ε) lineages, B.1.621 (Mu, μ), B.1.617.2 (Delta, δ) and very recently the introduction of B.1.1.529 (Omicron, o) (23). Here, we described a large RT-qPCR-based screening of mutations associated with known VOC/VOI/VUM, in SARS-CoV-2-positive samples obtained from patients seen at the emergency room and outpatients care services of the UC-Christus health network in Chile between January and November 2021. We validated our RT-qPCR-based assay with WGS in a subset of 636 samples, obtaining a 99.2% of concordance between both techniques. Additionally, all the data generated in this study was reported in real time to the local health authorities in our country.

## Methods

### SARS-CoV-2 Positive Samples

From January to November 2021, out of 4,782 RT-qPCR SARS-CoV-2 positives samples were obtained predominantly from symptomatic outpatients seen at the UC-Christus Health network and diagnosed in the Infectious Disease and Molecular Virology Laboratory. Briefly, 250 μL of nasopharyngeal swab resuspended in universal transport medium was obtained and processed by an automated extraction protocol using the Mag-Bind RNA Extraction Kit (Maccura Biotechnology CO., LTD) according to the manufacturer's instructions. The RT-qPCR assay for the detection of SARS-CoV-2 genome was performed with the LightMix^®^ SARS-CoV-2 RdRp-gene EAV PSR & Ctrl (TIB MOLBIOL) using 5 μL of extracted RNA as input and the final reactions were run in a LightCycler^®^ 480 real time-PCR system (Roche). The interpretation of the data was performed by analyzing the 2nd Derivative Maximum Method, obtaining the quantification cycle (Cq) value for each sample. Daily, we randomly selected samples that met the following criteria: being an emergency services consultant and having a RT-qPCR Cq of <32. We tested 1,851 samples, representing 38.7% of the total positive samples to determine the presence of mutations associated with VOC/VOI/VUM of SARS-CoV-2 by RT-qPCR. Samples originated mainly from Santiago (83.5%), the capital city, with a 16.5% of representation from the rest of the country. Subjects' mean age was 36.6, ranging from 2 days old to 98 years old, of which 50.38% were female. The subjects mean age in our study correlates with the mean age of SARS-CoV-2 infections in our country between January to November 2021 (24).

### Detection of Specific Mutations With RT-qPCR-Based Assays

The first screening was done by a multiplex RT-qPCR assay to detect mutations Del 69/70, E484K and N501Y in a single tube using the Allplex^TM^ SARS-CoV-2 Variants I assay (Seegene Technologies) according to the manufacturer's protocol in the Bio-Rad CFX96 qPCR instrument. A subsequent confirmation was performed by a second RT-qPCR that detected the P681H substitution using the VirSNiP SARS-CoV-2 Spike (TibMol Biol) or a custom TaqMan^®^ assay (Thermo Fisher Scientific) for the detection of P681H, K417T, and Del242-244. This workflow allowed us to discriminate in real time, between the mutations found at spike gene of SARS-CoV-2 associated with the Alpha (B.1.1.7), Beta (B.1.351), Gamma (P.1), Zeta (P.2), Eta (B.1.525), and Mu (B.1.621) variants. From June 2021 onwards, we extended to a newly available assays, where we additionally searched for mutations W152C, L452R, K417T, and K417N in a single tube (Allplex^TM^ SARS-CoV-2 Variants II assay, Seegene Technologies) to detect mutations associated with the Epsilon (B.1.427 and B.1.429) and Delta (B.1.617.2) variants. The introduction of new TaqMan^®^ assays (Thermo Fisher) in July 2021to detect L452Q and T478K mutations allowed us to suspect the presence of Lambda (C.37) and Delta variants, respectively. The turnaround time of the RT-qPCR-based screening until the final analysis is 3 h. The mutations associated with each variant used to discriminate between them are listed in Supplementary Table 1.

### RT-qPCR-Based Assays Validation With Next-Generation Sequencing

In a subset of samples with Cq between 13 and 32 (*n* = 636; 34.4%) the variant diagnosis after the RT-qPCR was validated by WGS by submission of 18 blinded aliquots to the Institute of Public Health (ISP, by the Spanish acronym) reference laboratory for sequencing through Illumina WGS technology using the Nextera DNA Flex Library Prep Kit in a MiSeq sequencer as detailed in (25). Other 618 samples were sequenced using the ARTIC SARS-CoV-2 ONT (Oxford Nanopore Technologies) sequencing protocol at the Molecular Virology Laboratory at PUC who employed the ARTIC V3/V4 whole-genome amplicon-based sequencing pipeline in a minION^TM^ Sequencer (Oxford Nanopore Technologies). Briefly, RNA extraction, cDNA synthesis and multiple PCR were done according to Tyson et al. (26). Then, libraries with 48–96 samples were performed according to the manufactures instructions (27) and for each sequence, the clade and lineage were identified according to the nomenclature of Nextstrain and Pangolin, respectively. Finally, complete genomes (>95% of coverage) were uploaded to GISAID. Derived data supporting the findings of this study are available from the corresponding author M.F on request.

### Statistical Analysis

Epidemiological and laboratory data are described as frequency (percentage, %) for categorical variables, and mean for quantitative variable data. Significative changes between frequencies were calculated using a *X*^2^ test with a 2x2 contingency table with GraphPad (Prism7, version 7.0a).

## Results

### RT-qPCR-Based Surveillance

**Figure 1:**
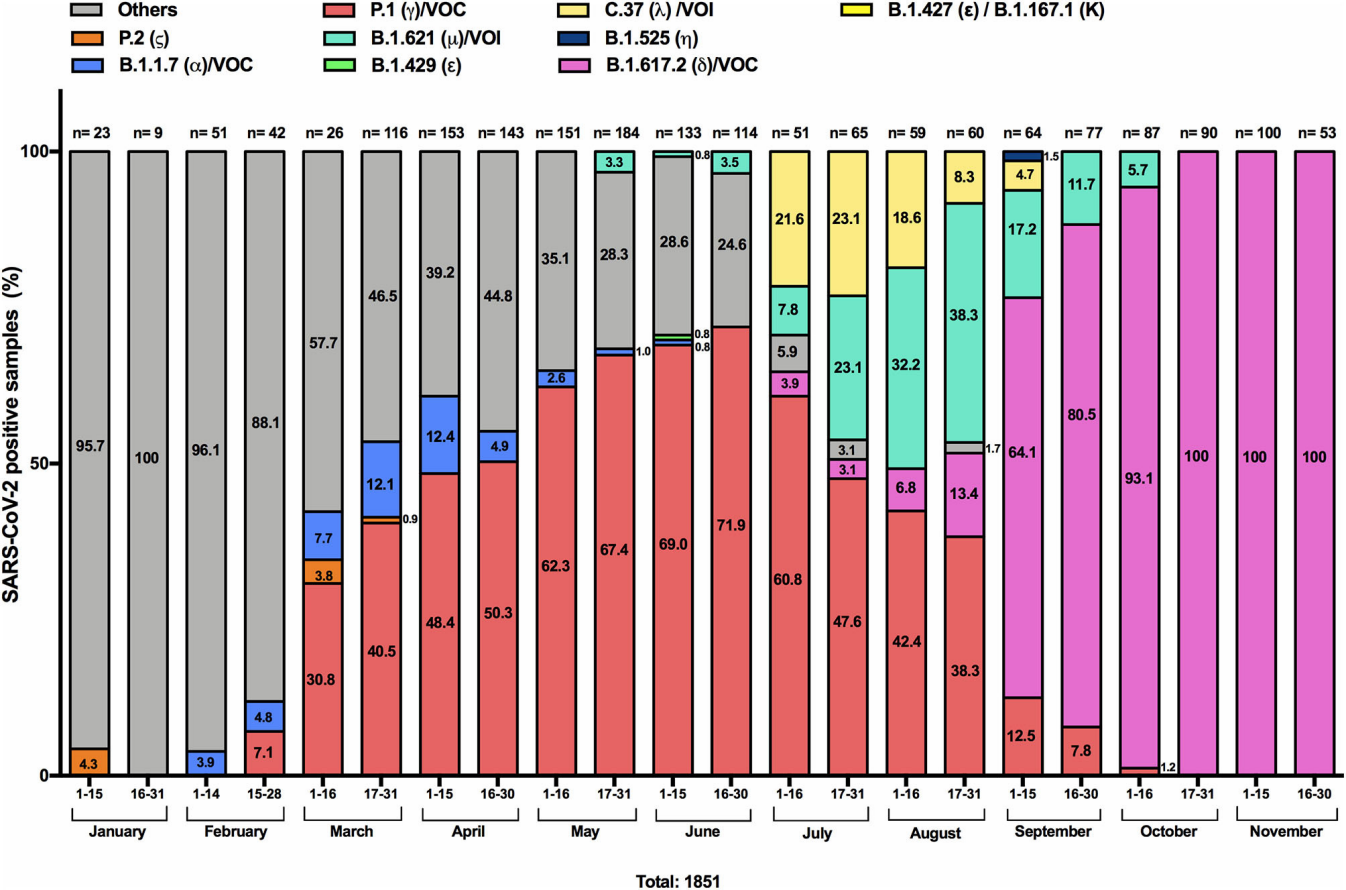
Proportion of SARS-CoV-2 positive samples with mutations associated with VOCs between January 1st and November 30, 2021 in Chile. Samples were screened by RT-qPCR assays aimed at identifying mutations: Del69/70, Del242-244, K417T, E484K, N501Y, P681H and W152C, K417N, L452R, L452Q, T478K. Samples that did not yield a positive signal for any of the examined mutations were classified as *others* (gray). Alpha (B.1.1.7; α) VOC (positive for Del69/70, N501Y, and P681H) is represented in blue; Gamma (P.1; γ) VOC (positive for: E484K, N501Y, K417T) in salmon color; Zeta (P.2; ζ) variant (positive only for E484K) in orange; Epsilon (B.1.429; ε) variant (positive for L452R and W152C) in green; Mu (B.1.621; μ) VOI (positive for: E484K, N501Y and P681H) in light green; Lambda (C.37; λ) VOI (Positive for: L452Q); Eta (B.1.525; η) variant in dark blue; and Delta (B.1.617.2 or A.Y lineages; δ) VOC in pink, are depicted. The data are presented in percentages, and the number of samples analyzed is indicated above each bar. The data are presented in percentages, indicating the number within each column. The total number of samples analyzed every 2 weeks is indicated above each bar.

To develop a rapid platform to identify the circulation of VOC, VOI and VUM in March 2021 we established RT-qPCR pipeline using commercially available kits based on mutations of known variants circulating worldwide. Additional assays were added to our analysis algorithm over time upon the emergence of new variants. Figure 1 depicts the frequencies of the respective identified variants from the total number of samples analyzed bimonthly, including retrospective analyses of samples from January-February 2021. The fraction resulting in undetermined variants (in gray color in Figure 1) was analyzed by WGS to obtain the virus genotype and determine additional mutations not identified by the available RT-qPCR assays. Through WGS we also confirmed the variant assignment and classified them as VOC, VOI and VUM in our surveillance cohort (Table 1). Using the RT-qPCR assays (Figure 1), in the first 2 weeks of January only one sample was identified with a mutation associated with the Zeta variant (P.2), representing 4.3% of the total samples analyzed. In February, mutations associated with the Alpha VOC (B.1.1.7) like Del69/70, N501Y, and P681H were identified in 3.9–4.8% of the samples and mutations (E484K, N501Y, K417T) associated with the Gamma VOC (P.1) in 7.1% of the samples tested the second half of February. From March onwards, we observed a significant increase (*p*-value: < 0.0001) in the presence of mutations associated with the Gamma variant, reaching a prevalence of 71.9% in the last 1 weeks of June (range 30.8–71, 9%). Toward the end of March and the first 2 weeks of April, the frequency of the Alpha variant peaked, reaching 12.4%, and then decreased over time until its disappearance during the first weeks of June. In June we were also able to identify one case with mutations (L452R and W152C) associated with the Epsilon variant (B.1.429), representing 0.8% of the total analyzed. Regarding the Lambda VOI, its circulation was retrospectively recognized through WGS from the beginning of February, and represented the major variant reported as others (in gray). This variant was not identifiable through the RT-qPCR assays until June, when specific probes were available. From July to September, the mutation associated with Lambda (L452Q), was observed at a frequency ranging from 21.6 to 4.7%. Similarly, our results indicate that from May, samples harboring the combination of mutations (E484K, N501Y, P681H) associated with the Mu VOI (B.1.621) appeared, to reach its peak (38.3%) during the last 2 weeks of August. Emergence of samples with the combination of mutations (L452R and T478K) associated with the Delta VOC (B.1.617.2) were detected since the first week of July. All samples associated with the Delta variant have been confirmed through whole genome sequencing. Noteworthy, Delta (L452R and T478K positive samples) rapidly became the predominant variant between August and November 2021, reaching 100% of all total samples analyzed since the last 2 weeks of October until the final date of the study in November 30th.

**Table 1:** Lineage assignment concordance between RT-qPCR-based screening and next-generation sequencing.

**OMS Name (Pango Lineage)**	Variants by RT-qPCR no/Total. (%)^*^	Variants by ILUMINA or Nanopore WGS no/Total. (%)^*^	**Main root mutations in S gene of SARS-CoV-2 lineages according to WGS^§^**
Alpha (B.1.1.7)^±^	18/636 (2.83)	18/636 (2.83)	**H69–**, **V70–**, **N501Y**, A570D, D614G, **P681H**, T716I, S982A.
Gamma (P.1)^±^	157/636 (24.69)	157/636 (24.69)	L18F, T20N, P26S, D138Y, R190S, **K417T**, **E484K**, **N501Y**, D614G, H655Y, T1027I, V1176F.
Delta (B.1.617.2)^±^	165/636 (25.94)	165/636 (25.94)	T19R, G142D, E156–, F157–, R158G, **L452R**, **T478K**, D614G, P681R.
Lambda (C.37)^¶^	34/636 (5.35)	29/636 (4.56)	R246–, S247–, Y248–, L249–, T250–, P251–, G252–, D253N, **L452Q**, F490S, D614G, T859N.
Mu (B.1.621)^¶^	63/636 (9.91)	63/636 (9.91)	T95I, Y144S, Y145N, R346K, **E484K**, **N501Y**, D614G, **P681H**, D950N.
Zeta (P.2)	2/636 (0.31)	2/636 (0.31)	**E484K**, D614G, Q677H, V1176F.
Epsilon (B.1.427/B.1.429)	1/636 (0.16)	1/636 (0.16)	S13I, **W152C**, **L452R**, D614G.
	**Total 440**	**Total 435**	
Unclassified by RT-qPCR	196/636 (30.81)	Not applicable	
Other lineages by WGS^*^
Lambda (C.37)^¶^^#^	Not Applicable	107/636 (16.82)	
B.1.1.348	Not Applicable	42/636 (6.60)	
N.4	Not Applicable	14/636 (2.20)	
Others	Not Applicable	38/636 (5.97)	
Total	636	636	

* *Percentage of other lineages calculated from the total number of samples analyzed*.

# *Before the RT-qPCR L452Q assays were available*.

± *VOC, Variant of Concern*.

¶ *VOI, Variant of Interest*.

§ *Panel of mutations available for RT-qPCR assays are depicted in bold font*.

### Assays Validation With WGS

To validate our RT-qPCR-based surveillance workflow for the assignment of the presence of known SARS-CoV-2 variants, a portion of SARS-CoV-2 positive samples was derived blindly for real-time WGS analysis. Of the total (1,851 samples analyzed), 636 were sequenced through either the Illumina platform or the ARTIC SARS-CoV-2 ONT (Oxford Nanopore Technologies) WGS protocols. Table 1 summarizes the results of VOC/VOI/VUM identification based on RT-qPCR and WGS. Samples harboring mutations associated with Alpha (*n* = 18), Gamma (*n* = 157), Delta (*n* = 165), Mu (*n* = 63), Zeta (2) and Epsilon (B.1.429, *n* = 1) were validated through whole genome sequencing in the 100% of the samples. In the diagnosis of the Lambda variant, there was a disagreement with WGS, in which five samples were diagnosed as B.1.1.348. This inconsistency was because the identification of this variant was done based on only one mutation (L452Q), which is also shared by other variants. Hence, in this case confirmation of Lambda will required an additional RT-qPCR (not currently available) or WGS. In summary, our result showed a 99.2% agreement (631/636) between RT-qPCR-based diagnosis and WGS analysis, suggesting that our established RT-qPCR assays provide a rapid and accurate algorithm for identifying variants.

## Discussion

The emergence of new variants of SARS-CoV-2 and their possible implications in the COVID-19 disease is of great concern to public health authorities worldwide (2, 4, 16, 28). Genomic surveillance through WGS is crucial for the identification of new variants. However, many laboratories around the world, especially in underdeveloped or developing countries, do not have the sequencing capacity or resources to scale genomic surveillance to provide real-time information (28). Knowing the local epidemiology context, and the new variants that have emerged over time, we sought to determine specific mutations of the known SARS-CoV-2 variants using assays based on RT-qPCR starting from the same RNA sample used for SARS-CoV-2 diagnosis, lowering the cost and time of variant identification. This rapid and straight forward methodology can be developed in the same diagnostic laboratories and would allow large-scale surveillance in low-and-middle incomes countries (17, 28). As a proof of concept, in this study we performed a screening using commercially available assays that aimed to identify the mutations Del69/70, E484K, N501Y, P681H, Del242-244, W152C, K417T, K417N, L452R, L452Q, T478K, which allowed us to discriminate, based on presence/absence of these mutations, among the VOC/VOI/VUM that have circulated in the country (23). The proposed workflow was validated by whole genome sequencing and lead us to quickly identify the Zeta, Alpha, Gamma, Epsilon (B.1.429), Lambda, Mu, Eta and Delta variants and to suspect the presence of Kappa or Epsilon (B.1.427). The data presented here is in agreement with the reports issued from local health authorities about variant circulation (through genomic surveillance) in Chile during the same period (23). The data obtained indicate that in the first trimester of 2021, the circulation of the Lambda VOI was predominant according to the WGS data. From February to July, we observed the rapid emergence of mutations associated with the Gamma variant (P.1), period coinciding with a second wave of infections and an increase in hospitalizations due to COVID-19 in Chile (29). In our country, the introduction of the P.1 lineage was detected on January 30th, 2021, in a traveler arriving from Brazil (23). On June 25, 2021, the ISP laboratory through genomic surveillance reported 1,479 cases (43.0%) of the Gamma (P.1) variant, followed by 837 cases (24.3%) of Lambda (C.37) variant (23). These samples derived from individuals from different regions of our country, ambulatory cases, outbreaks, re-infected individuals and travelers (23). Our work was focused on symptomatic outpatients of a Health Network in Metropolitan region, where approximately one third of Chilean population lives. Hence, the small differences in the proportion of the P.1 lineage compared to our study can be explained by differences on sampling selection and the population they represent. Nevertheless, the major representation and consistent increase of P.1 lineage between March and June, are in close agreement in both reports (23). With our screening based in RT-qPCR, we were able to monitor and report in real-time the appearance of a combination of mutations associated with the Mu variant since May 2021, increasing in circulation and displacing the Gamma and Lambda variants. Nonetheless, Gamma, Lambda, and Mu variants were rapidly replaced by samples harboring a combination of mutations associated with the Delta VOC (B.1.617.2). The rise in the identification of Delta variant was consistent with the genomic surveillance data through WGS updated in November 29th by the local Chilean authorities (23). Importantly, our assays were 99.2% consistent when compared with results obtained by whole genome sequencing, and therefore, they represent an important tool for outbreak surveillance in order to carry out a timely containment of community infection.

To our knowledge, the present study is the first report of surveillance through RT-qPCR-based approaches in Chile. Our results demonstrate that RT-qPCR is a useful and complementary tool to quickly and efficiently determine the presence of mutations associated with VOC/VOI/VUM in different clinical settings in real time. As well as other studies (12, 17, 21, 22, 28), we propose a RT-qPCR-based screening to search for specific mutations of SARS-CoV-2 lineages to carry out a large-scale surveillance, leaving the samples unidentifiable by this methodology as a priority to be sequenced through WGS. This approach offers the advantage of working with high-throughput methods in the same facility where the diagnosis is conducted and uses the same extracted RNA used for diagnosis, reducing the cost per assay. Despite the encouraging results, the interpretation should be taken with caution and should be adapted and validated constantly as the emergence of new circulating variants must be confirmed by WGS locally (28). In addition, the appearance of new mutations that could have the potential to interfere with diagnostic tests must be checked constantly.

## Data Availability Statement

The datasets presented in this study can be found in online repositories. The names of the repository/repositories and accession number(s) can be found below: GISAID.

## Ethics Statement

The studies involving human participants were reviewed and approved by Institutional Ethical Review Board of Pontificia Universidad Católica de Chile (code: 210615005). Written informed consent from the participants' legal guardian/next of kin was not required to participate in this study in accordance with the national legislation and the institutional requirements.

## Author Contributions

JA, CM-V, NL, and MF designed the study. JA, CM-V, EF-L, AC, and CM performed the RT-qPCR experiments and basic statistical analysis. CP-R, LA, RM, and FM performed the WGS experiments and bioinformatic analysis. JA, CM-V, and MF wrote the manuscript. All authors contributed to the article and approved the submitted version.

## Funding

This work was supported by the Agencia Nacional de Investigación y Desarrollo (ANID) of Chile (FONDECYT N° 1211825 to MF, CM-V, and JA, N° 11180167 to JA, COVID0920 to NL and CM-V, N° 3190706 to CP-R); and DIDEMUC SC-11 to MF, JA, CM-V, and NL. Work at the Medina Laboratory was partially funded by the FONDECYT N° 1212023 grant, the Center for Research on Influenza Pathogenesis (CRIP), an NIAID Center of Excellence for Influenza Research and Surveillance (CEIRS, contract # HHSN272201400008C), the NIH-NIAID 1U19AI135972 grant.

## Conflict of Interest

The authors declare that this study received funding from BH Chile INC to conduct SARS-CoV-2 genomic surveillance. The funder was not involved in the study design, collection, analysis, interpretation of data, the writing of this article or the decision to submit it for publication.

## Publisher's Note

All claims expressed in this article are solely those of the authors and do not necessarily represent those of their affiliated organizations, or those of the publisher, the editors and the reviewers. Any product that may be evaluated in this article, or claim that may be made by its manufacturer, is not guaranteed or endorsed by the publisher.
