# Supplementary material for: Assessment of Mutations Associated With Genomic Variants of SARS-CoV-2: RT-qPCR as a Rapid and Affordable Tool to Monitoring Known Circulating Variants in Chile, 2021
Source: Front Med (Lausanne). 2022 Feb 25;9:841073. doi: 10.3389/fmed.2022.841073 (PMC8914012; doi:10.3389/fmed.2022.841073)
Supplement: Supplementary file 1 [file Table_1.DOCX]

**Supplementary Table 1:** Panel of available RT-qPCR assays used in the study to identify mutations and deletions of SARS-CoV-2 (columns) and interpretation of the associated lineages (rows) based on the positive (+) or negative (-) amplification.

|  |  | **Mutations in S gene of SARS-CoV-2** | | | | | | | | | | |
| --- | --- | --- | --- | --- | --- | --- | --- | --- | --- | --- | --- | --- |
|  |  | **Del69-70** | **Del242-244** | **W152C** | **K417N** | **K417T** | **L452R** | **L452Q** | **T478K** | **E484K** | **N501Y** | **P681H** |
| **Variants of Concern*** | **Alpha**  B.1.1.7 (20I) | **+** | **-** | **-** | **-** | **-** | **-** | **-** | **-** | **-/+** | **+** | **+** |
|  | **Beta**  B.1.351 (20H) | **-** | **+** | **-** | **+** | **-** | **-** | **-** | **-** | **+** | **+** | **-** |
|  | **Gamma**  P.1 (20J) | **-** | **-** | **-** | **-** | **+** | **-** | **-** | **-** | **+** | **+** | **-** |
|  | **Delta**  B.1.617.2 (21A) | **-** | **-** | **-** | **-** | **-** | **+** | **-** | **+** | **-** | **-** | **-** |
|  | **Omicron**  B.1.1.529 (21K) | **+** | **-** | **-** | **+** | **-** | **-** | **-** | **+** | **-** | **+** | **+** |
| **Variants under monitoring** | **Zeta**  P.2 (20B) | **-** | **-** | **-** | **-** | **-** | **-** | **-** | **-** | **+** | **-** | **-** |
|  | **Eta**  B.1.525 (20A) | **+** | **-** | **-** | **-** | **-** | **-** | **-** | **-** | **+** | **-** | **-** |
|  | **Epsilon**  B1.427 (20C) | **-** | **-** | **-** | **-** | **-** | **+** | **-** | **-** | **-** | **-** | **-** |
|  | **Epsilon**  B.1.429 (20C) | **-** | **-** | **+** | **-** | **-** | **+** | **-** | **-** | **-** | **-** | **-** |
|  | **Lambda**  C.37 (21G) | **-** | **-** | **-** | **-** | **-** | **-** | **+** | **-** | **-** | **-** | **-** |
|  | **Mu**  B.1.621 (21H) | **-** | **-** | **-** | **-** | **-** | **-** | **-** | **-** | **+** | **+** | **+** |
|  | **Kappa**  B.1.167.1 (21B) | **-** | **-** | **-** | **-** | **-** | **+** | **-** | **-** | **-** | **-** | **-** |
|  | **Delta PLUS**  B.1.617.2.1 (21A) | **-** | **-** | **-** | **+** | **-** | **+** | **-** | **+** | **-** | **-** | **-** |

*Variant nomenclature according to WHO (bold font), Pangolin (normal font) and Nextstrain (parenthesis).
